# Rapid referral for headache management from emergency department to headache centre: four years data

**DOI:** 10.1186/s10194-020-01094-6

**Published:** 2020-03-14

**Authors:** Andrea Negro, Valerio Spuntarelli, Paolo Sciattella, Paolo Martelletti

**Affiliations:** 1 Department of Clinical and Molecular Medicine, Sapienza University, Rome, Italy; 2 Regional Referral Headache Centre, Sant’Andrea Hospital, Rome, Italy; 3 Department of Statistical Sciences, Sapienza University of Rome, Rome, Italy

**Keywords:** Headache, Migraine, Non-traumatic headache, Emergency department

## Abstract

**Background:**

Headache is one of the most common reason for medical consultation to emergency department (ED). The inappropriate use of ED for non-emergency conditions is a problem in terms of overcrowding of emergency facilities, unnecessary testing and treatment, increased medical costs, burden on medical service providers and weaker relationships between patient and primary care provider. The aim of this study was to analyze the different stages of ED management of headache to identify those deficiencies that can be overcome by a fast referral to a headache clinic.

**Methods:**

The study is a retrospective analysis of the electronic medical records of patients discharged from an academic ED between January 1, 2015 and December 31, 2018 and referred to the tertiary level headache centre of the same hospital. We analyzed all aspects related to the permanence in ED and also assessed whether there was a match between the diagnosis made in ED and ours.

**Results:**

Among our sample of 244 patients, 76.2% were admitted as “green tag”, 75% underwent a head computed tomography, 19.3% received a neurological consultation, 43% did not receive any pharmacological treatment and 62.7% still had headache at discharge. The length of stay in ED was associated with reporting the first aura ever (*p* = 0.014) and whether patients received consultations (*p* < 0.001). The concordance analysis shown a significant moderate agreement only for the diagnosis of migraine and only between triage and headache centre.

**Conclusions:**

Most patients who went to ED complaining of headache received the same treatment regardless of their diagnosis and in many cases the headache had not yet resolved at the time of discharge. Given the many shortcomings in headache management in ED, rapid referral to the headache centre is of paramount importance to help the patient achieve a definiteve diagnosis and appropriate treatment.

## Introduction

Headache is one of the most common symptoms and an important reason for medical consultation and presentation to emergency departments (EDs). In fact, of all patients visiting EDs, up to 4.5% report that non-traumatic headache is their main complaint [1]. The third edition of the International Classification of Headache Disorders (ICHD-III) distinguishes between secondary headaches, where headache is a symptom, and primary headaches, which include migraine (with or without aura), tension-type headache and the group of trigeminal autonomic cephalalgias (TACs) for which cluster headache counts for the majority [2]. Migraine alone is the world’s second leading cause of years of life lived with disability [3] and chronic migraine (CM) patients, who have ≥15 headache days per month, are more likely to have a higher disability, lower quality of life and greater use of healthcare resources than patients with episodic migraine (EM) [4, 5].

In the ED, as in the general population, primary headache disorders are much more common than secondary disorders and it can be challenging for ED doctors to distinguish the few patients with potentially life-threatening headaches from the vast majority with benign headaches [6]. Primary headache is diagnosed in 58–81.2% of ED patients with headache and, for the specific diagnosis of primary headache, migraine is the main condition, representing 17–64% of cases [7–11]. While most headaches have a benign etiology and are self-limiting, up to 5% have a serious and life-threatening cause (e.g., subarachnoid hemorrhage) that requires immediate medical attention [12, 13].

Inappropriate use of ED for non-emergency conditions is a problem in terms of overcrowding of emergency facilities, unnecessary testing and treatment, increased medical costs, burden on medical service providers and weaker relationships between patient and primary care provider. Studies evaluating this phenomenon have found that the inability to take time off from school or work during the day and the ease of use of emergency care without the need for an appointment are cited as a reason for their consultation by a significant percentage of patients, suggesting that inappropriate use of ED could be a matter of personal convenience [14, 15]. An observational study investigated the factors associated with ED visits for headache and found that the most common reason for visiting ED was a perceived emergency condition or referral by a physician (33.3%) [16]. The other most commonly cited reasons were the timeliness of the treatment, either because the doctor’s office was closed (20%) or because patients could not get an appointment early enough (11%), or because they did not have a doctor or no other place to go (9%) [17]. Studies conducted in other countries suggest that primary headaches are under- or misdiagnosed and, as a result, patients are often undertreated or receiving inadequate care when consulting an ED [8, 17, 18].

In our hospital there is close collaboration with ED and all patients suffering from headache after discharge can be referred to our clinic and visited within 36 h.

Given the lack of Italian studies on patients presenting with headache to ED, we analyzed the different stages of headache management to identify the deficiencies that can be overcome with a quick referral to a headache clinic. In particular, we evaluated the demographic and clinical features of these patients, the frequency and accuracy of headache diagnosis, the treatment they received and their follow-up in our headache center.

## Methods

The study is a retrospective analysis of the electronic medical records (EMRs) of patients who presented to the academic ED of Azienda Ospedaliera Sant’Andrea, between January 1, 2015 and December 31, 2018, with a chief compliant of headache and who, after discharge, were referred to the Regional Referral Headache Centre, Department of Clinical and Molecular Medicine, Faculty of Medicine and Psychology, Sapienza University of Rome.

### Data collection

The data analyzed included basic demographic information, presentation time, priority assessment and diagnosis of admission by the hospital triage nurse, door-to-doctor time and the length of stay in ED, life parameters, diagnostic investigations, specialist consultations, treatment administration and discharge diagnosis. We also assessed the time elapsed between discharge from the ED and the first visit to our headache center and whether there was a match between the diagnosis of discharge from the ED and ours.

For priority admission, our ED uses an advanced triage system that provides a color-coding scheme that uses white, green, yellow and red tags to define conditions at an increasing level of severity:

- white tags - (dismiss) are given to those with minor injuries for whom a doctor’s care is not required;
- green tags - (wait) are reserved for the “walking wounded” who will need medical care at some point, after more critical injuries have been treated;
- yellow tags - (observation) for those who require observation (and possible later re-triage). Their condition is stable for the moment and, they are not in immediate danger of death. These victims will still need hospital care and would be treated immediately under normal circumstances;
- red tags - (immediate) are used to label those who cannot survive without immediate treatment but who have a chance of survival.

For vital signs analysis, elevated blood pressure (BP) was defined as follows: systolic BP (SBP) ≥140 mmHg or diastolic BP (DBP) ≥90 mmHg [19].

The diagnosis in our headache centre was made on the basis of the beta version or the final version of the ICHD-III depending on the year in which the visit was carried out [2, 20].

### Statistical analyses

A descriptive analysis of the sample characteristics was carried out. The categorical data were summarized by numbers and percentages, the continuous data by mean and standard deviation. The association between total time in ED and follow-up at the headache centre was evaluated by Mann-Whitney tests. The concordance between primary headaches diagnosis was assessed using the Cohen Kappa coefficient (κ). The significance level was set at 0.05 (always corrected if necessary). All analyses were performed using SAS 9.4 (SAS Institute Inc., Cary, NC, USA).

## Results

### Demographics and clinical characteristics of the study population

Demographic and clinical characteristics of patients treated in ED are summarized in Table 1. The sample analyzed consisted of 244 patients, 192 (78.7%) women and 52 (21.3%) men, aged between 14 and 89 years and an average of 40.7 years. After evaluating the general conditions, the triage nurses coded one patient (0.4%) as a white tag, 186 (76.2%) as a green tag, 56 (23.0%) as a yellow tag and one patient (0.4%) as a red tag. Medical history was negative for any conditions in 157 (64.3%) patients. Among the various pathologies, the most frequent were hypertension (in 11.1% of patients), psychiatric disorders (in 8.6% of patients) and thyroid diseases (in 7.0% of patients). Patients presented to ED after an average of 1.9 days after the onset of the headache for which they needed assistance. The appearance of a migraine aura was the main reason for consulting for ED for 40 (16.4) patients and for 16 (40%) of them it was the first aura of their lives. Mean values of vital signs for the whole sample of patients were in the normal range: systolic pressure was 130 mmHg (range: 100–190), diastolic pressure was 77 mmHg (range: 50–111), heart rate was 78 bpm (range: 39–116), temperature was 36.6 °C (range: 35.9–38) and oxygen saturation was 98% (range: 94–100).

**Table 1:** Demographic and clinical characteristics of patients treated in the emergency department

*Demographics*	
age (years) (SD)	40.7 (16.3)
sex (female)	192 (78.7)
*Medical history*	
negative	157 (64.3)
hypertension	27 (11.1)
psychiatric disorders	21 (8.6)
thyroid disorders	17 (7.0)
hematologic disorders	10 (4.1)
dyslipidemia	9 (3.7)
DM type 2	8 (3.3)
cerebrovascular diseases	7 (2.9)
COPD/asthma	4 (1.6)
ischemic heart disease	3 (1.2)
solid tumor	3 (1.2)
*Time of ED presentation*	
08:00–15:59	147 (60.3)
16:00–23:59	79 (32.4)
00:00–07:59	18 (7.4)
*Headache onset*	
days bedore ED visit	1.9 (5.7)
*Triage codes*	
white	1 (0.4)
green	186 (76.2)
yellow	56 (23.0)
red	1 (0.4)
*Vital signs*	
systolic BP (mmHg)	130 (16.9)
diastolic BP (mmHg)	77 (10.6)
heart rate (bpm)	78,910.60
temperature (°C)	36.6
oxygen saturation (%)	98 (1.4)
*Aura*	
reason to access ED	40 (16.4)
first aura in their life	16 of 40 (40.0)
*ED times in min, mean (SD)*	
door-to-doctor time	139 (118.4)
time of care	241 (269.8)
ED length of stay	381 (307.7)

*DM* diabetes mellitus, *ED* emergency department, *SD* standard deviation

### Diagnosis of headache in the emergency department

Table 2 summarized the changes in diagnoses in relation to the setting of the clinical evaluation. We found eleven different admission diagnoses assessed by the triage nurse. The three most common diagnoses were headache (63.6%), migraine without aura (9.0%) and ophthalmic headache (6.6%). ED physicians used fewer diagnoses (four) and the most frequent was headache (52.5%).

**Table 2:** Changes in diagnoses in relation to the clinical evaluation setting

DIAGNOSIS	TRIAGE nurse	ED doctor	Headache centre	Concordance TRIAGE - headache centre
	*(244 patients)*	*(244 patients)*	*(240 patients)*	*(κ; p - value)*
Headache	155 (63.6)	128 (52.5)	–	
Migraine without aura	22 (9.0)	82 (33.6)	140 (57.4)	0.581; *p* < 0.001
Migraine with aura (ophthalmic headache)	16 (6.6)	–	34 (13.9)	0.032; *p* = 0.586
Tension-type headache	–	–	32 (13.1)	–
Chronic migraine	–	–	35 (14.3)	–
Medication overuse headache	–	–	31 (12.7)	–
Cluster hedache	2 (0.8)	2 (0.8)	7 (2.9)	−0.013; *p* = 0.001
Other TACs	–	–	1 (0.4)	–
Trigeminal neuralgia	8 (3.3)	–	3 (1.2)	–
Headache attributed to trauma	5 (2.1)	32 (13.1)	8 (3.3)	–
Sinusitis	–	–	2 (0.8)	–
Headache attributed to cranial and/or cervical vascular disorder	16 (6.6)	–	6 (2.5)	–
Headache and arterial hypertension	10 (4.1)	–	5 (2.0)	–
Headache and systemic infection	6 (2.5)	–	1 (0.4)	–
Headache and anxiety disorder	3 (1.2)	–	–	–
Cervicogenic headache	1 (0.4)	–	–	–

*TACs* trigeminal autonomic cephalalgias

Triage nurses used the definition “ophthalmic headache” to indicate the diagnosis of “migraine with aura”

The concordance between primary headaches diagnosis was assessed using the Cohen Kappa coefficient (κ). The significance level was set at 0.05

There was not agreement between diagnoses made by ED physicians and headache centre

### Headache investigations received in the emergency department

Most patients performed complementary examinations for diagnosis (Table 3). The most common investigation was head computed tomography (CT) required for 183 (75%) patients. One patient with a known diagnosis of migraine refused to have a CT scan. Of the 182 CT scans performed 171 (94%) were negative and 11 (6%) had findings that were all considered incidental: sphenoid and maxillary sinus mucosal thickening (3), mega cisterna magna (2), sinusitis (1), chronic ischemic leukoencephalopathy (1), < 5 mm engagement of cerebellar tonsils into the foramen magnum (1), subcutaneous sebaceous cyst (1), aneurysm of basilar artery (1) and occipital bone exostoses (1). The second most common investigation was electrocardiogram (ECG) performed in 31 (12.7%) patients without any correlation with vital signs. All ECGs were reported as non-pathological. Finally, 16 (6.6%) patients underwent other investigations besides head CT and ECG: supra-aortic trunk echo-doppler (8), sinus CT scan (3), brain magnetic resonance imaging (MRI) (2), cervical X-rays (2) and electroencephalogram (1).

**Table 3:** Interventions in the emergency department

*Investigations*	
head CT	183 (75.0)
ECG	31 (12.7)
other	16 (6.6)
*Specialist consultations*	
neurologist	47 (19.3)
ophthalmologist	7 (2.9)
otolaryngologist	5 (2.0)
psychiatrist	5 (2.0)
cardiologist	2 (0.8)
neurosurgeon	2 (0.8)
*Treatment administered*	
none	105 (43.0)
NSAIDs	108 (44.3)
weak opioids	43 (17.6)
antiemetics	31 (12.7)
PPIs	25 (10.2)
paracetamol	20 (8.2)
corticosteroids	16 (6.6)
anxiolitics	12 (4.9)
antibiotics	2 (0.8)
*Classes of drugs (in ED)*	
one	61 (25.0)
two	45 (18.4)
three	24 (9.8)
four	7 (2.9)
five	2 (0.8)

*CT* computed tomography, *ECG* electrocardiogram, *ED* emergency department, *NSAIDs* non-steroidal anti-inflammatory drugs, *PPIs* proton pump inhibitors

### Specialist consultations received in the emergency department

Most patients were managed by ED physicians without the need for specialist consultation (Table 3). Of a total population of 244 patients, 47 (19.3%) were examined by a neurologist, 7 (2.9%) by an ophthalmologist, 5 (2.0%) by an otolaryngologist, 5 (2.0%) by a psychiatrist, 2 (0.8%) by a cardiologist and 2 (0.8%) by a neurosurgeon**.**

### Headache treatment received in the emergency department

Pharmacological treatments administered in ED are summarized in Table 3. NSAIDs and weak opioids were the most commonly prescribed pharmacological agents, being administered in 44.3% and 17.6%. of patients respectively. None of the 244 patients received a triptan. Treatment of associated symptoms included antiemetics and anxiolytics, administered to 12.7% and 4.9% of patients respectively. Proton pump inhibitors were administered to 10.2% of patients. Approximately 43% of patients received no pharmacological treatment, while among patients treated 25% received monotherapy, and 18.4% and 9.8% received a combination of two or three pharmacological agents, respectively.

### Triage and stay information in the emergency department

Information on ED stay of the 244 patients is summarized in Table 4. The median door-to-doctor time was 139 (range: 3–881), the median time of care (from first contact with ED to discharge) was 241 min (range: 1–1404) and the mean time of stay (from arrival in ED to discharge) was 381 min (range: 20–2212). There was no significant correlation between the length of stay in ED and a number of factors (arrival time in ED, triage coding, blood pressure, head CT scan, ECG, other investigations and treatment administration). In contrast, the length of time spent in ED was significantly associated with some factors. In particular, having received neurological consultation (*p* < 0.001) or other specialist visits (*p* = 0.001) was associated with a longer stay in ED while the complaint of the first aura ever (*p* = 0.014) was associated with a shorter stay in ED.

**Table 4:** Factors that influence the length of stay in the emergency department

LENGTH OF STAY IN ENERGENCY DEPARTMENT					
	mean	min	max	SD	*p-value*
Time of arrival					
08:00–15:59	381	20	2212	327	NS
16:00–23:59	393	37	1540	277	
00:00–07:59	321	72	740	211	
Triage					
white	320	320	320	.	NS
green	380	20	1540	282	
yellow	387	37	2212	373	
red	202	202	202	.	
Aura					
no	368	20	2212	291	NS
yes	446	37	1412	360	
First aura					
no	527	133	1412	376	0.014
yes	325	37	1386	306	
SBP ≥ 140 mmHg					
no	383	20	2212	304	NS
yes	373	37	1529	306	
DBP ≥ 90 mmHg					
no	389	20	2212	312	NS
yes	271	92	479	114	
HEAD CT					
no	349	20	1540	270	NS
yes	391	37	2212	314	
ECG					
no	359	20	1540	260	NS
yes	531	37	2212	492	
Other investifgations					
no	372	20	2212	294	NS
yes	510	37	1386	413	
Neurological consultation					
no	333	20	2212	252	< 0.001
yes	580	37	1533	408	
Other consultations					
no	359	20	2212	279	0.001
yes	625	115	1533	447	
Treatment					
no	385	41	1529	303	NS
yes	377	20	2212	305	

*CT* computed tomography, *DBP* diastolic blood pressure; *NS* not significant, *SBP* systolic blood pressure, *SD* standard deviation

### Discharge

Discharge information are summarized in Table 5. At the time of discharge from ED the headache had not disappeared in 62.7% of the 244 patients and 43.9% of the patients had been discharged without a prescription. The most commonly prescribed medications for the treatment of headache were NSAIDs and paracetamol, administered to 33.2% and 11.1% of patients respectively, while triptans were prescribed to only 1.6%. Monotherapy was prescribed more frequently than combination therapy with two agents (44.7%% vs 10.2%). Only 5.8% of patients were prescribed prophylactic treatment upon discharge. The most common post-discharge investigation was cerebral MRI, which was prescribed to 4.1% of patients, while in 92.6% of patients no further investigation was required.

**Table 5:** Discharge from the emergency department

*Headache at discharge*	
no	153 (62.7)
yes	91 (37.3)
*Drugs prescription*	
none	107 (43.9)
NSAIDs	81 (33.2)
paracetamol	27 (11.1)
weak opioids	19 (7.8)
prevention	14 (5.8)
corticosteroids	5 (2.0)
triptans	4 (1.6)
anxiolitics	2 (0.8)
antiemetics	1 (0.4)
antibiotics	1 (0.4)
*Classes of drugs (at discharge)*	
one	109 (44.7)
two	25 (10.2)
three	3 (1.2)
*Post-discharge investigations*	
brain MRI + angio-MRI	12 (4.8)
EEG	2 (0.8)
psychiatric visit	2 (0.8)
Holter blood pressure	2 (0.8)

*EEG* electroencephalogram, *MRI* magnetic resonance imaging, *NSAIDs* non-steroidal anti-inflammatory drugs

### Follow-up in the headache Centre

Information on follow-up visit to the headache centre is summarized in Table 6. The average follow-up time at our headache centre was 8.9 days (range: 1–30) after ED discharge and 4 (1.6%) patients did not show up for the appointment. Considering the entire sample of 244 patients, 97.5% were new patients and 2.5% were already followed up at our headache clinic. All 240 patients who attended the follow-up appointment at our clinic were diagnosed with headache according to the ICHD [2, 20]. Most patients (66.7%) were already aware of the diagnosis. A primary form of headache was diagnosed in 90% of patients, while a secondary headache was diagnosed in 10%. The three most common primary headache diagnoses were migraine without aura (53.3%), CM (14.6%) and migraine without aura (14.2%). The three most common diagnoses of secondary headache were medication overuse headache (MOH) (12.7%), headache attributed to trauma (3.3%) and headache attributed to cranial and/or cervical vascular disorder (2.5%). A double diagnosis was given to 24.6% and a triple diagnosis to 1.2% of the patients. The most frequent combinations of two diagnoses were CM and MOH (11.7%) followed by migraine with aura and migraine without aura (9.6%). The most frequent combination of three diagnoses was CM, MOH and migraine with aura (0.8%) followed by CM, MOH and headache attributed to cranial and/or cervical vascular disorder (0.4%). The average age at the onset of their headache was 25 years (range: 6–89) and 45% had a family history of migraine. Patients were managed according to their headache diagnosis. All patients with a new migraine diagnosis were prescribed specific acute treatment for migraine (e.g., triptans), where it was not contraindicated. Those patients with a known diagnosis of migraine who had already experienced problems of efficacy and/or tolerability with the triptan they had already used, were prescribed another molecule belonging to the triptan class and/or analgesic combination (e.g., paracetamol plus caffeine or codeine). Both patients with migraine and tension-type headache with > 4 attacks per month received oral prevention treatments according to international guidelines [21]. With regard to CM prevention, 24 (68.6%) of 35 patients with this diagnosis were prescribed quarterly injections of onabotulinumtoxinA [22, 23] and 26 (83.9%) of 31 patients with MOH started an in-patient withdrawal and rehabilitation protocol [24].

**Table 6:** Follow-up in the headache centre

Age at the onset (years) (SD)	25 (18.1)
New patient to our headache clinic	238 (97.5)
Days since discarge	8.9 (7.2)
Familiarity for headache (yes)	108 (45.0)
Aware of their diagnosis (yes)	160 (66.7)
Headache days per month	12.6 (9.5)
OnabotulinumtoxinA for CM	24 of 35 (68.6)
Rehabilitation for MOH	26 0f 31 (83.9)

*CM* chronic migraine, *MOH* medication overuse headache, *SD* standard deviation

## Discussion

We conducted a retrospective study on a consecutive sample of 244 patients observed in our tertiary headache centre after direct referral from the ED of our hospital, where they presented with a chief compliant of headache. Our aim was to analyzed the different stages of ED management of patients with headache to identify those deficiencies that can be overcome by a rapid referral to a headache clinic.

The first step of this journey is the need to make the correct diagnosis. The assessment of headache patients in the ED environment differs significantly from the assessment of headache patients in the clinic. Management of headache in an acute setting should focus on the exclusion of serious secondary causes, correct diagnosis, symptoms management and possibly the establishment of a continuing care plan [25]. The sense of urgency to exclude secondary and often life-threatening causes of headache, also due to time constraints due to the need for efficient patient management, may lead ED physicians to interrupt the diagnostic process on the differential diagnosis between primary and secondary headache. A Canadian study to assess the diagnostic accuracy of neurological complaints in ED found that in 35.7% of cases the initial ED diagnosis was not consistent with the final diagnosis made by a neurologist; among the most common misdiagnosed problems were primary headaches [26]. There was a high rate of discrepancy between the diagnosis made in our headache centre and the diagnosis made in ED. This discrepancy may have several reasons. ED physicians may be more interested in excluding serious causes of headache than in distinguishing different primary headaches or they are simply not familiar with the diagnostic criteria of ICHD. However, even if they were aware of the diagnostic criteria, ED physicians may not have adopted them because they intended to use the same treatment regardless of the diagnosis of primary headache. In addition, there is a possibility that ED physicians may simply accept the diagnosis of a triage nurse or not change the diagnosis already in the system. However, the interesting finding was that triage nurses used eleven different diagnosis, most of which simply describing a condition associated with headache, while ED physicians used only four diagnostic categories (Table 2). This finding provides further evidence of what ED physicians may consider to be their primary task, namely to rule out severe secondary headache rather than make a definitive diagnosis. The analyses of concordance among the three different diagnoses (triage, ED physician and headache centre) showed a significant moderate concordance only for the diagnosis of migraine and only between triage and headache centre. This latter finding is perhaps due to the fact that most patients (66.7%) were already aware of their headache diagnosis and the triage nurse took the information obtained from the patients and used it to compile the triage diagnosis.

The second step of the journey are the diagnostic investigations that may be necessary to confirm the diagnostic suspect and establish consistent treatment. Head CT scans are commonly used to assess headache due to their rapid accessibility and diagnostic accuracy, but unnecessary head CT scans lead to a longer ED stay [27], exposure to radiations [28] and increased medical costs [29]. The use of CT scans for non-traumatic headache in the United States EDs has doubled in the last 20 years [30]. Up to 31% of patients who had headache underwent imaging [30] and head CT scans accounted for almost 50% all CT scans in the United States EDs [31]. However, almost 95% of the CT scans carried out in the United States EDs between 1992 and 2001 showed no pathological results [12]. We found that 75% of patients who attended our ED with a major compliant of headache had a CT scan, which was negative in 93.4% of cases, while in the other cases the results were all considered accidental and not life-threatening (Table 3). However, this large use of CT scan in ED requires serious reflection. In fact, a recent study found that a 9.6% decrease in the use of head CT scans for patients presenting to ED with a major headache complaint was not followed by an increase in deaths or missed diagnoses [32] This data provides convincing evidence that there is a possibility of safely decreasing CT scans for ED patients. Conversely, in support of CT use, another study found that patients who underwent a head CT scan during an initial ED visit were about half as likely to return to ED within 30 days than those who had not had CT [33]. Head imaging has been shown to have a positive influence on patients’ fear and anxiety levels [34] and, by reassuring patients, CT scan prevent them from returning to ED. However, we can hypothesize that a rapid referral to a headache unit may have, at least in some patients, a similar reassuring effect, but avoiding the impact of the CT scan on the patient and the healthcare system.

The third step is the request for specialist consultations to add experience to the work of ED physicians. In our ED at least one neurologist is available 24 h a day, 7 days a week, both for the first line assessment if the pre-hospital assessment suggests a neurological condition, and for the second line, according to the judgement of a ED physician who is not a neurologist when the patient arrives. However, less than a fifth (19.3%) of patients received a neurological consultation (Table 3). This rate was lower than in another study in French EDs, where one third of patients received a consultation with a neurologist [8]. The authors hypothesized that this may have been due to the fact that patients were experiencing an unusually severe attack or because a differential diagnosis was considered necessary for patients who had reported a first episode of headache. We can speculate that there may be a number of reasons for the fewer consultations in our study, including the doctor’s certainty that a secondary headache is ruled out and knowledge of the possibility of a rapid referral to our headache clinic. Further consultations were requested for 8.2% of patients and the three most frequently consulted specialists were ophthalmologists (for aura), otolaryngologists (for sinusitis) and psychiatrists (for comorbid conditions). However, receiving specialist consultations, both neurological and other, was the only factor that was significantly associated with a longer stay in ED (Table 4). On the contrary, the complaint of the first aura in life was associated with a shorter stay in ED. It is possible that when faced a patient who reported having previously had other episodes of aura, the ED doctor was reassured on the benignity of the symptom, while in the case of the first aura the doctor was more cautious and felt the urgency to handle the case. This fact could also indicate an inadequate knowledge of the diagnostic criteria of primary headaches in general, and specifically the clinical spectrum of migraine.

The fourth and fundamental step in the management of headaches in ED is the pharmacological treatment. Patients with migraine or other headaches have the third highest self-reported pain score of all patients presenting to ED with a painful condition [35]. Consistently, the American Migraine Prevalence and Prevention (AMPP) study found that unbearable pain was the most common reason for the use of ED for migraine [36]. Patients attending ED with a headache pattern similar to previous migraine attacks generally do not require, or do not want, diagnostic tests, but expect rapid and effective management of their headache and any misdiagnosis can lead to generalized pain treatment rather than of specific treatment for migraine [18]. More than 20 different drugs and drug combinations are used to treat migraine in ED, including migraine-specific drugs (e.g., sumatriptan and dihydroergotamine), dopamine antagonists (e.g., metoclopramide, chlorpromazine and prochlorperazine), NSAIDs, opioids, corticosteroids, and anti-histamines (e.g., diphenhydramine and promethazine) [37]. In our ED, the most commonly prescribed pharmacological agents were NSAIDs and weak opioids, administered to 44.3% and 17.6%. of patients respectively, followed by paracetamol, which was administered to 8.2% of patients (Table 3). Treatment of the associated symptoms included antiemetics and anxiolytics, administered to 12.7% and 4.9% of patients respectively. Another study conducted in Europe showed opposite use of NSAIDs (42.9%) and non-opioid analgesics (61.2%) and lower use of antiemetics (8%) and anxiolytics (3%) [8]. In the above mentioned study, approximately 9% of patients did not receive any pharmacological treatment and triptans were not administered frequently (11.2%) [8]. In our sample, about 43% of patients did not receive any medications and no triptans (or ergotamines) were administered. However, the underutilization of headache medications in ED is not limited to European countries. A survey conducted in a university hospital ED in the United States found an equally high percentage of patients (38%) who received neither drugs nor intravenous fluids [18].

The role of the ED physician is not only to rule out a secondary headache disorder but to provide adequate headache treatment; but the latter goal is not always accomplished. In our study, headache had not disappeared at the time of ED discharge in 62.7% of the 244 patients. This rate, dramatic in itself, was still lower than that of another study in the French department, where migraine had not disappeared at the time of discharge from ED in 80.2% of patients [8]. There are significant variations in headache management between and within EDs due to lack of strong recommendations, physician comfort and familiarity with specific medications, belief in efficacy, concern about short-term side effects, and patient demand [37–39]. Only recently, the Canadian Headache Society and the American Headache Society have provided evidence-based therapeutic recommendations for migraine requiring treatment in emergency settings [40, 41].

The fifth and final step in the management of headaches in ED is discharge. Significant evidence suggests that migraine discharge management is often suboptimal. About 40% of emergency physicians never prescribed triptans on discharge [42], about 60% of patients had no documented discharge medications and 2/3 of patients did not receive a medical follow-up recommendation [18]. These malpractices contribute to the high rate of ED return visits noted among migraine patients [43]. Another study found a high percentage of patients (34.7%) discharged without a prescription and again the most commonly prescribed drugs were analgesics and NSAIDs rather than migraine-specific drugs [8]. Our data, in line with the previous literature, showed that 43.9% of patients were discharged without a prescription, while the most commonly prescribed drugs for the treatment of headache were NSAIDs (33.2%) and paracetamol (11.1%), while triptans were prescribed only to 1.6% (Table 5).

Considering what has been discussed earlier on the journey through ED of a patient complaining of headache, a fast track between ED and a headache clinic is a step forward in headache management. Headache is the most common disorder among patients presenting to ED with neurological complaints [44] and although in most cases it is discharged as a non-urgent disorder, these patients feel they need urgent medical evaluation [45]. On the contrary, migraine patients, especially those with a known diagnosis, should have the idea that their headache is not a life-threatening condition and if they get to ED it is more because of access problems and less because of a perceived need for emerging treatment. Inappropriate use of ED leads to overcrowding and consequently an increase in intervention times, which is one of the main reasons for leaving ED prematurely [46], especially among patients with a compliant of headache [47]. This suggests that a healthcare system could reduce ED visits among migraineurs by increasing the availability of urgent care or walk-in appointments [48].

Further evidence of the importance of rapid referral to a headache centre comes from an analysis of ED use by migraine patients. The AMPP study showed that only a small subset of respondents used ED for the treatment of severe headaches in the previous 12 months: 3% visited the ED once and another 3% visited ED more than once [36]. Interestingly, in a previous study, we showed that among the 548 migraine patients who attended our headache clinic and were undergoing continuous treatment and regular follow-up visits, only 0.9% of them entered the ED during the entire 2-years observation period [49]. This is explained by the fact that migraine prevention and prescription of migraine-specific rescue therapies reduce the use of resources in general, and ED visits in particular [50–52]. In addition to improving the health and quality of life of patients, a preferential route between ED and the headache clinic can lead to significant cost savings, considering that ED care is considerably more expensive than both hospital outpatient care and office-based outpatient care. At a time of economic hardship and availability of new and expensive treatments, such as monoclonal antibodies targeting the calcitonin gene-related peptide, a virtuous allocation of economic resources becomes mandatory [53].

## Limitations

The study has several potential limitations. First, there are inherent limitations related to the use of the data collected primarely for clinical care, such as errors in recording some information that could be perceived as secondary importance in the particular setting of ED. However, we believe that the variables of primary interest in this analysis, including socio-demographic characteristics, diagnostic procedures, specialist consultations and drugs should not have been subject to inaccurate recording. Secondly, the enrollment period was between January 1, 2015 and December 31, 2018 and, for this reason, most patients were diagnosed according to the beta version of the ICHD-III published in 2013, while the definitive version was published in early 2018. Thirdly, the decision of ED physicians whether or not to refer a patient discharged from the ED for a severe headache to our headache centre is totally personal and independent. As a result, there is a possibility that patients may have been referred mainly by some physicians and not by others and that this may introduce a selection bias towards one type of patient rather than another. Finally, this study was conducted in a single university hospital for tertiary care and our results may not be generalized to other countries or even to other Italian regions or to other EDs that may have different organizational and therapeutic protocols.

## Conclusions

The vast majority of patients who went to the emergency room complaining of a headache received the same treatment regardless of the cause of the headache and for this reason in many cases the headache had not yet resolved at the time of discharge. We have shown a high rate of misdiagnosis of primary headaches and a short route between ED and the headache center is of primary importance to help the patient achieve a definitive diagnosis and appropriate treatment. Reducing the number of emergency visits for primary headaches, and in particular migraine headaches, is the goal and easy and rapid referral to headache specialists can prevent further recourse to ED. In addition, an implementation of referral from ED to the headache center of the same hospital, if present, can increase the quality of patient management and, by sharing information about hospital’s EMRs, can avoid the need to repeat diagnostic investigations and reduce time and expense, thus leading to a better allocation of public resources.

## Data Availability

Dataset available from the corresponding author on reasonable request.

## Abbreviations

BP: Blood pressure
CGRP: Calcitonin gene-related peptide
CM: Chronic migraine
CT: Computed tomography
ECG: Electrocardiogram
ED: Emergency department
EEG: Electroencephalogram
EM: Episodic migraine
EMR: Electronic medical records
ICHD: International classification of headache disorders
MOH: Medication overuse headache
MRI: Magnetic resonance imaging
NSAIDs: Non-steroidal anti-inflammatory drugs
TACs: Trigeminal autonomic cephalalgias
